# Effect of Climatic Factors and Population Density on the Distribution of Dengue in Sri Lanka: A GIS Based Evaluation for Prediction of Outbreaks

**DOI:** 10.1371/journal.pone.0166806

**Published:** 2017-01-09

**Authors:** PDNN Sirisena, Faseeha Noordeen, Harithra Kurukulasuriya, Thanuja ALAR Romesh, LakKumar Fernando

**Affiliations:** 1 Department of Microbiology, Faculty of Medicine, University of Peradeniya, Peradeniya, Sri Lanka; 2 Department of Earth Sciences, Postgraduate Institute of Science, University of Peradeniya, Peradeniya, Sri Lanka; 3 Department of Mathematics, Faculty of Science, University of Peradeniya, Peradeniya, Sri Lanka; 4 Centre for Management of Dengue and Dengue Haemorrhagic Fever, General Hospital, Negombo, Sri Lanka; National Taiwan Ocean University, TAIWAN

## Abstract

Dengue is one of the major hurdles to the public health in Sri Lanka, causing high morbidity and mortality. The present study focuses on the use of geographical information systems (GIS) to map and evaluate the spatial and temporal distribution of dengue in Sri Lanka from 2009 to 2014 and to elucidate the association of climatic factors with dengue incidence. Epidemiological, population and meteorological data were collected from the Epidemiology Unit, Department of Census and Statistics and the Department of Meteorology of Sri Lanka. Data were analyzed using SPSS (Version 20, 2011) and R studio (2012) and the maps were generated using Arc GIS 10.2. The dengue incidence showed a significant positive correlation with rainfall (*p*<0.0001). No positive correlation was observed between dengue incidence and temperature (*p* = 0.107) or humidity (*p* = 0.084). Rainfall prior to 2 and 5 months and a rise in the temperature prior to 9 months positively correlated with dengue incidence as based on the auto-correlation values. A rise in humidity prior to 1 month had a mild positive correlation with dengue incidence. However, a rise in humidity prior to 9 months had a significant negative correlation with dengue incidence based on the auto-correlation values. Remote sensing and GIS technologies give near real time utility of climatic data together with the past dengue incidence for the prediction of dengue outbreaks. In that regard, GIS will be applicable in outbreak predictions including prompt identification of locations with dengue incidence and forecasting future risks and thus direct control measures to minimize major outbreaks.

## Introduction

The global prevalence of dengue has increased dramatically in recent decades with 390 million people getting infected annually [1]. In Sri Lanka the highest dengue incidence is seen in the Western Province with 55% (26153/47502) of the total number of cases occurring in 2014 and 33% (9881/29777) of the total number of cases occurring in 2015 [2]. The total number of reported dengue incidences were relatively less in 2015, however, there has been a dramatic increase in the dengue incidence in other provinces due to high rainfall mainly in the Southern, North Central and Uva resulting in less percentage of cases in the Western Province in 2015 [2,3]. In the first half of 2016, there has been an alarming increase in the dengue incidence in the whole country including almost all the provinces. Moreover, in the recent past, the dengue incidence has been more marked in the Northern Province as the disease has spread to the province with the movement of people after the cessation of war in 2009/2010 with 5.4% (2564/47502) of the total number of cases occurring in 2014 vs 0.3% (47/15551) of the total number of cases occurring in 2004 [2]. The Sabaragamuwa Province had a dengue incidence of 9.6% (4565/47502) in 2014 compared to 6% (933/15551) in 2004. These data highlight the potential threat posed by severe dengue epidemics in Sri Lanka in the future [2].

Sri Lanka is an island nation positioned between 5° and 10° north latitude experiencing a tropical warm climate, moderated by ocean winds with considerable moisture. The mean temperature in the country ranges from a low of 15.8°C in Nuwara Eliya in the Central Highland to as high as 29°C in Trincomalee on the North Eastern coast. The average annual temperature of the country ranges from 26–28°C [3]. The country gets rain from two monsoons: the South West monsoon prevails from April to September and the North East monsoon prevails from December to February. In between there are inter-monsoons in March to April and a second inter-monsoon in October and November [4]. Levels of humidity range from 60–90% during different seasons. The humidity is high in the South West mountainous areas and the humidity changes with rainfall.

The risk associated with dengue and severe dengue is well known and felt in Sri Lanka due to the disease burden on the country’s developing economy [5], however, the impact of climate factors on the increase in the disease incidence and occurrence of outbreaks need detailed exploration in relation to different climatic zones and districts in the island. Hence, identification and mapping the geographical areas in different climatic zones and districts at risk and predicting future dengue outbreaks will contribute to better prevention of dengue throughout the country.

There is direct and indirect influence of climate in the disease transmission, distribution, vector breeding and establishment [6]. Previous studies have shown that climate change and global warming are responsible for the expansion of arboviral diseases in the world [7,8]. Vector breeding, mortality, behaviour, viral replication within the mosquito vector and the time required for dengue virus (DENV) to become transmissible to another host after initial infection in a mosquito are temperature dependent [9,10,11,12]. Scott et al (2000) [13] shows the effect of temperature on the vector population dynamics starting from the development of egg to immature mosquitoes [9]. Moreover, the ovarian development and survival at different stages of the life cycle of the vector is influenced by temperature [9]. Temperature and rainfall work inter-dependently with each other influencing the vector dynamics and thus temperature indirectly influences the rainfall by regulating the evaporation. These then have an influence on the availability of water habitats for the *Aedes* vector breeding.

The relationship between rainfall and dengue outbreaks has been shown in previous studies [14,15,16]. Rainfall contributes to create and maintain breeding sites for vectors. GIS and climate data analysis done in Hawaii show intra-annual climate variations inducing changes in *A*. *albopictus* habitats [17]. High dengue incidence is associated with areas of less rainfall and warmer temperatures as reported by a Caribbean study. The same study also provides evidence for well-defined seasonality with dengue epidemics. Moreover, temporal distribution of dengue cases is shown to be associated with pre-rainfall periods [18]. A Sri Lankan study conducted in the Western Province provides evidence for a strong correlation between dengue outbreaks and pre-rainfall [19].

High humidity results from high rainfall combined with high temperatures. High humidity is associated with increased feeding activity, survival and development of eggs in *A*. *aegypti*. Moreover, the daily minimum temperature and an increase in the rainfall from the previous month were associated with increase in the larval abundance [20]. Thus there is a collective contribution of temperature and humidity on dengue outbreaks triggered by the feeding activity, survival and development of vectors [21].

Previous studies help to predict vector borne disease epidemics in relation to climate factors using GIS mapping. GIS, remote sensing and spatial statistics will allow researchers to identify the patterns of dengue incidence in a country and will help to evaluate the association between dengue incidence and climatic factors [22]. GIS mapping would help in planning and controlling the vectors in high-risk areas and to institute vector control measures. GIS mapping is one of the less focused areas in Sri Lanka at present and this needs to be explored to predict and forecast outbreaks. Efforts are needed to train relevant personnel to effectively utilize and apply remote sensing and related technologies for controlling dengue, which drains the developing economy.

Thus the objective of the current study was to use GIS methodology to map and evaluate the spatial and temporal distribution of dengue in Sri Lanka from 2009 to 2014 and to elucidate the association of geographical and climatic risk factors with dengue incidence. We have evaluated the spatial and temporal distribution of dengue with geographical and climatic risk factors for 12 districts representing all 3 agro-climatic zones including wet, dry and inter-mediate zones in the country.

## Materials and Methods

### Study area

Ethical approval for the research project was obtained from the Ethical Review Committee, Faculty of Medicine, University of Peradeniya (2011/EC/49) before starting the project. However, this study did not use patients or specimens collected from patients and only used data from different sources as stated.

Sri Lanka is divided in to 9 provinces and 25 districts. To represent all the provinces at least one or two districts from each province was/were selected as follows: for the Northern Province (NP), Jaffna District; North Western Province (NWP), Puttalam and Kurunegala Districts; Western Province (WP), Colombo District; North Central Province (NCP), Anuradhapura District; Central Province (CP), Kandy and Nuwara Eliya Districts; Sabaragamuwa Province (SGP), Ratnapura District; Eastern Province (EP), Trincomalee and Batticaloa Districts; Uva Province (UP), Badulla District: Southern Province (SP), Hambantota District. These12 districts represent 3 agro-climatic zones, wet, dry and inter-mediate zones of Sri Lanka (Table 1).The current study focuses on the effect of climatic factors on dengue incidence and outbreaks in different agro-climatic zones using data for selected districts situated in these agro-climatic zones.

**Table 1:** Meteorology stations according to the agro-climatic zones,elevation above sea level (given in metres (m)) andglobal positioning system (GPS) coordinates oflatitude and longitude for each meteorological station (DD) (Meteorology Department of Sri Lanka, 2006).

Wet zone			Dry zone			Intermediate zone		
District	*E (m)	Latitude | Longitude **DD	District	*E (m)	Latitude | Longitude **DD	District	*E(m)	Latitude | Longitude **DD
Colombo—WP	7	6.927079|79.861243	Puttalam—NWP	2	8.040791|79.839386	Kurunegala—NWP	116	7.472981|80.354729
Ratnapura -SGP	34	6.705574|80.384734	Trincomalee—EP	3	8.587364|81.215212	Badulla—Uva	670	6.993401|81.054981
Kandy—CP	479	7.33599|80.6214	Hambantota—SP	16	6.142883|81.121231			
Nuwara Eliya—CP	1880	6.949717|80.789107	Anuradhapura—NCP	93	8.311352|80.403651			
			Jaffna—NP	10	9.661498|80.025547			
			Batticaloa—EP3	3	7.730997|81.67473			

*E (m)–Elevation in meters

**DD—Decimal degrees.

### Data collection

The dengue disease data was collected from the Epidemiology Unit, Ministry of Healthcare and Nutrition, Sri Lanka. The Epidemiology Unit releases epidemiological data quarterly for different districts and these data are useful for a chronographic and geographic evaluation of dengue. Population data were obtained from the Census and Statistic Report, Department of Census and Statistics, Sri Lanka (2012) as this was the latest Census and Statistic Report available for the country. Annual rainfall, temperature and humidity data for Sri Lanka collected from the Meteorology Department and GPS of meteorological stations (latitude and longitude) are given in Table 1.

### Data analysis

The data were statistically analyzed using the SPSS, Version 20 (2011) and R studio, (2012) software. The spatial distribution of dengue incidence and climatic factors were mapped using the Arc GIS, Version 10.2 (2012) software.

## Results

**Fig 1:**
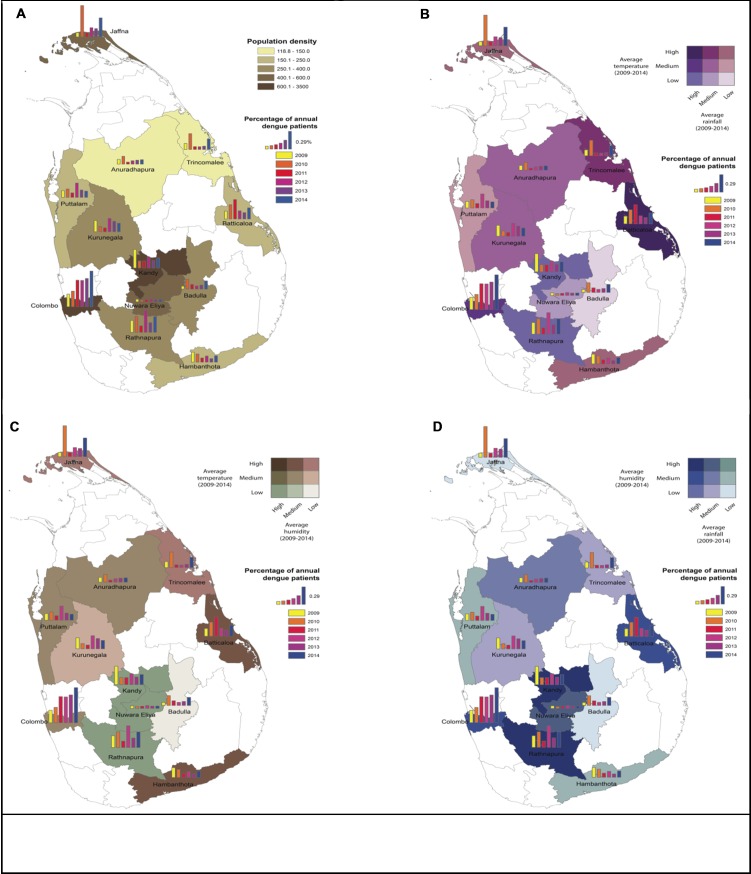
Correlation between total annual dengue incidence with climatic factors and population density. Correlation of annual dengue incidence from 2009–2014 with population density (A), temperature and rainfall (B), temperature and humidity (C) and humidity and rainfall (D).

A few GIS studies have been conducted to identify the transmission dynamics of dengue in Sri Lanka and these studies were limited to a few districts. Sri Lanka is divided into 9 Provinces, 25 Districts and the current study has evaluated data from 12 Districts covering all three agro-climatic zones. The dengue incidence was high in areas where the population density is high and this was clearly seen in the three major Districts, Colombo, Kandy and Jaffna where the population density is high. In 2009, Sri Lanka experienced a dengue outbreak of unprecedented magnitude affecting the whole island. In 2010, the country experienced another major outbreak affecting mainly Anuradhapura, Badulla, Batticalo, Puttalam, Ratnapura, Trincomalee and Jaffna Districts. In the past six years, there has been a persistent increase in the reported number of dengue cases in the Colombo District when compared to other Districts where there have been minimal variations in the number of reported cases. Even with high population density in the Nuwara Eliya, the District shows has had a very few dengue cases in the past six years (Fig 1A). Apart from Nuwara Eliya, all other Districts in the wet zone namely Colombo, Ratnapura and Kandy reported a higher dengue incidence. In the dry zone, Districts with high population such as Batticola, Puttalam and Jaffna also showed high incidence of dengue. Moreover, Kurunegala District with high population density in the intermediate zone also showed high dengue incidence. Changes in dengue incidence with temperature and rainfall, temperature and humidity and rainfall and humidity from 2009–2014 in 12 Districts were mapped and shown in Fig 1B–1D.

The correlation between dengue incidence and climatic factors including temperature, rainfall and humidity was evaluated using the Spearman’s correlation, which was considered as significant at a level of 0.01. An increase in the average rainfall had a positive correlation with dengue incidence (*p*<0.0001). However, there was no positive correlation noted between dengue incidence and temperature (*p* = 0.107) or dengue incidence and humidity (*p* = 0.084).

A time series analysis was performed to identify the serial correlations and trends in the data over time, seasonal variations in the data over time and the possibility of forecasting future dengue outbreaks. To predict the correlation of dengue incidence vs humidity, dengue incidences vs rainfall and dengue incidence vs temperature, a correlogram was plotted. A collerogram is useful for stationary time series data and thus to evaluate the non-stationary time series data, typical trends and periodicities were removed before investigating the auto-correlational structures in the dataset.

**Fig 2:**
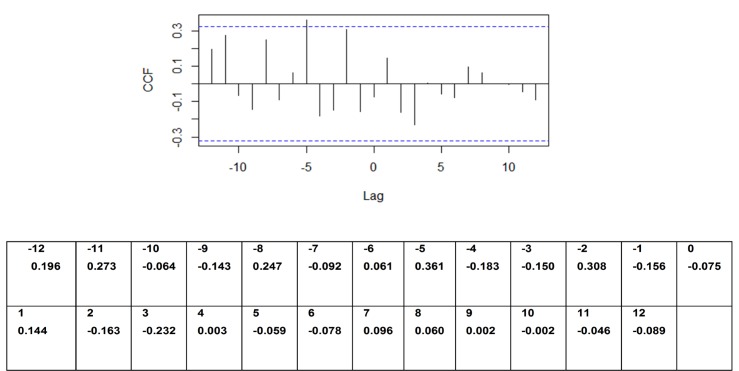
Auto-correlation of series ‘X’ by lag–dengue vs rainfall. Y axis–CCF- Cross correlation function; X axis–Lag time in months.

**Fig 3:**
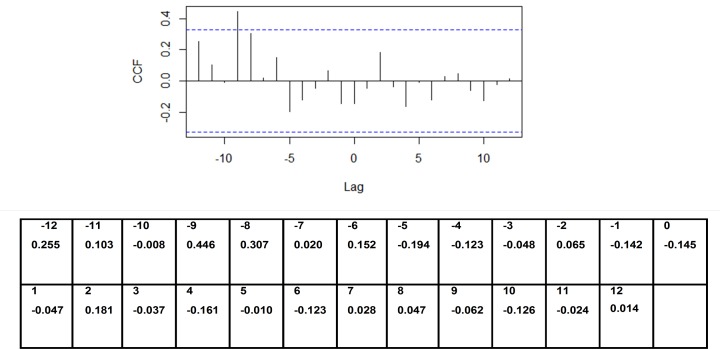
Auto-correlation of series ‘X’ by lag–dengue vs temperature. **Y** axis–CCF—Cross correlation function; X axis–Lag time in months.

**Fig 4:**
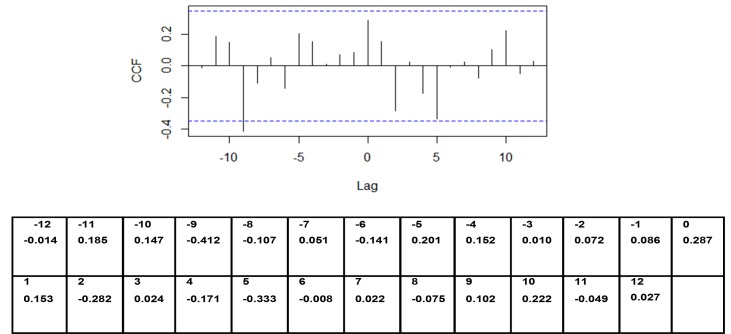
Auto-correlation of series ‘X’ by lag–dengue vs humidity. Y axis–CCF—Cross correlation function; X axis–Lag time in months.

According to the auto-correlation analysis there was a strong positive correlation noted between dengue incidence and an increase in rainfall prior to 5 months from the increase in dengue incidence. There was a weak positive correlation noted with an increase in rainfall prior to 2 months from the increase in dengue incidence (Fig 2). A significant positive correlation between dengue incidence and an increase in temperature prior to 9 months from the increase in dengue incidence was also noted (Fig 3). A mild positive correlation between dengue incidence and humidity was observed with a rise in humidity prior to 1 month, although a significant negative correlation was observed with a rise in humidity prior to 9 months from the increase in dengue incidence (Fig 4).

**Fig 5:**
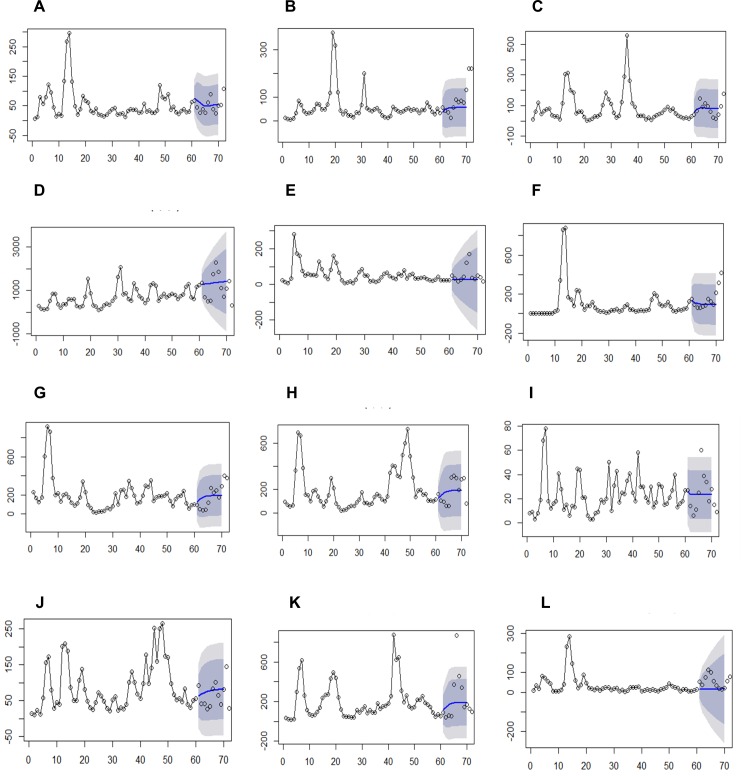
ARIMA models for 12 districts. A—Anuradapura; B—Badulla; C—Batticaloa; D—Colombo; E -Hambantota; F—Jaffna; G—Kandy; H—Kurunegala; I–Nuwara Eliya; J—Puttalam; K—Ratnapura and L—Trincomalee Districts.

To predict future outbreaks in 12Districts, an autoregressive integrated moving average (ARIMA) model was generated. This model is an explicit statistical model for the irregular component of a time series data that allows non-zero auto-correlations in irregular components in the dataset. Dengue incidence data was collected from 2009 to 2014. Using data up to 2013, dengue incidence were predicted for 2014 to evaluate the accuracy of forecasting. All data points for 2014 fell within the forecasted area allowing the model to be evaluated for the prediction of future outbreaks (Fig 5).

**Fig 6:**
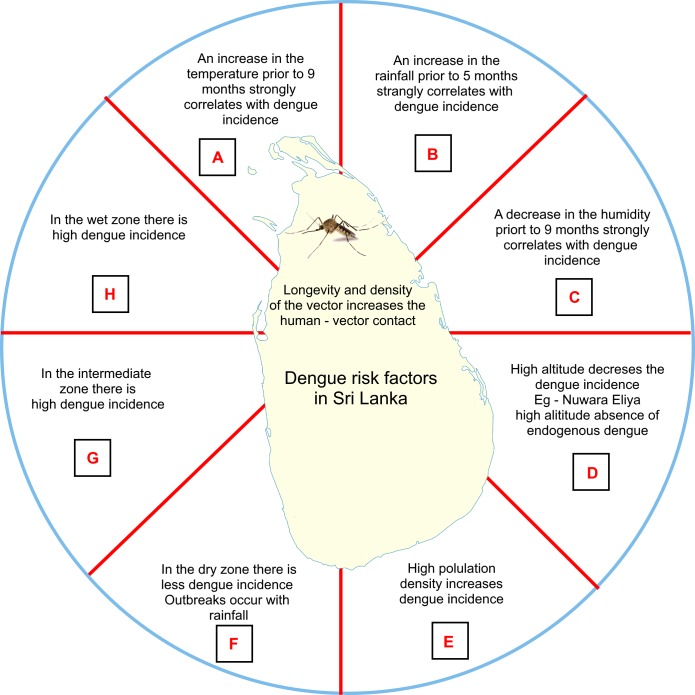
A dashboard showing dengue risk factors in Sri Lanka with contingency measures for dengue control. A, B and C—Authorities must follow the changes in the temperature, rainfall and humidity and direct control efforts to the risk areas based on the changes in these climatic factors; D—Be vigilant and prevent the introduction of vectors to the District; E—Better city construction and urban development, maintaining proper drainage facilities and solid waste management with inspection for construction rules and legislations; F- Regular and continuous inspection of water collection practices in the households and directing control measures immediately after the seasonal rainfall; G and H—Be vigilant throughout the year and direct control measures before and after the rains- pre-rainfall cleaning will help to remove the water collection bodies in the area minimizing the vector habitats and post rain fall cleaning up will further eliminate the vector hidden breeding sites.

Using the summary of results obtained from this study, a dashboard was generated to show the dengue risk in Sri Lanka. A feasible control strategy is also proposed considering the risk factors in different agro-climatic zones (Fig 6).

## Discussion

Dengue has become endemic in the whole island of Sri Lanka with infections occurring in epidemic proportions in all Districts and Provinces, making a huge burden on the country’s health care system and in turn to the growing economy. This study was designed to identify the dengue risk associated with population density, land pattern and climatic factors using a GIS based evaluation to design a dashboard to assist health authorities to promptly spot high-risk areas to initiate control activities.

The Colombo District from the Western Province has been experiencing a very high dengue incidence and the highest number of cases reported from 2009–2014 was from this District. The major reason behind the high number of reported cases in the Colombo District appears to be due to the high population density [23,24]. A study [23] done during the current study period also suggests an increase in the reported cases from Jaffna (NP) and Batticaloa (EP) and the total number of cases from these Districts might have dominated even the total reported cases from Colombo in 2010. One of the reasons for the increase in the cases from Jaffna and Batticaloa might also be increased population density in these districts as free travel allowed movement of people following the end of war in 2009/2010 [23]. Taken together these findings, an increased population density contributes to an increase in dengue incidence as noted in several highly populated Districts of Sri Lanka such as Colombo, Kandy, Jaffna and Batticaloa [5,24]

The dengue incidence is very low in the Nuwara Eliya District despite this being one of the highly populated District in the wet zone. High altitude seems to play a pivotal role in limiting the distribution of *A*. *aegypti* in the Nuwara Eliya District which is situated at 1880 m above the sea level. Badulla District is situated in the next highest elevation of 670 m and has a lower population density than Nuwara Eliya, yet reported high dengue incidence in the last 5 years. In India, *A*. *aegypti* breeding sites range from the sea level to 1000 m above the sea level. Lower elevations (<500 m) have moderate to heavy mosquito populations, while mountainous areas (>500 m) have low mosquito populations [25] supporting the inverse association between higher elevation and the vector activity and thus less dengue incidence as seen in Nuwara Eliya.

With two monsoon seasons in Sri Lanka, the absolute number of dengue and severe dengue cases peak twice annually. The first peak occurs in June/July that coincides with the South Western monsoon due to the rainfall that starts in April and the second peak occurs at the end of the year to early January next year associated with the North Eastern monsoon that occurs from October to December (S1 Fig) [4]. This confirms our auto-correlation data in time series analysis that rainfall prior to two months is responsible for biannual peaks of dengue incidence. A previous study [26] using the weekly rainfall data in the country showed that the total rainfall slightly influences dengue incidence in Colombo and Anuradhapura. However, the current study points out the impact of the rainfall prior to 2 months from the outbreak with increased dengue incidence. Moreover, rainfall prior to 5 months also strongly correlates with dengue incidence. In contrast to these findings, a previous study conducted in the Matara District of the Southern Province related less rainfall and relative humidity and higher temperature with high dengue incidence during 2010–2012 [27]. The explanation for high dengue incidence with less rainfall might be that *A*. *aegypti* transmitted outbreaks are caused by breeding indoor in the manmade breeding sites. Dengue incidence is low during heavy rainfall but increases when the rainfall started to decrease, showing a 3–4 weeks lag time between the heavy rainfall and dengue outbreaks [19] and this is also true during floods and heavy rain falls at different times of the year in different agro-climatic zones of the country.

The average rainfall had a positive correlation with dengue incidence (*p*<0.0001) suggesting the contribution of high rainfall with increased dengue incidence. Apart from the Nuwara Eliya District that is situated in the highest altitude, all the other districts including the Colombo District in the wet zone experienced increased dengue incidence throughout the study period and the wet zone receives the highest rainfall in the country.

Districts representing intermediate and dry zones show occasional outbreaks in par with rainfall and water storage practices as also noted by an Indian study [28]. In some Southeast Asian countries where the annual rainfall is more than 200 cm, *A*. *aegypti* populations are more stable and established in urban, semi-urban and rural areas. However, in countries like Indonesia, Myanmar and Thailand, the mosquito densities are shown to be higher in semi-urban areas than in urban areas due to the traditional water storage practices used in the semi-urban areas [29]. In contrast to the strong positive correlation between dengue incidence and the rainfall pattern in many tropical countries, dengue outbreaks occur before the arrival of the rainy season or in relatively dry seasons in some parts of the world due to the availability and expansion of mosquito-breeding sites resulting from water storage measures used in dry seasons. Conversely, in certain countries like Singapore, a positive correlation between rainfall and vector density has not been observed as the country experiences rain throughout the year [30]. In countries where there are two distinct rainy seasons, a positive correlation has been observed in only one season [31] and not in the other. Although clear reasons cannot be identified for the discrepancies in dengue incidence or outbreaks in different countries of the tropics with rainfall, it might be possible that the impact of rainfall on adult vector density is not the same for all vector species. *A*. *aegypti* prefers indoor habitats, hence, it is less affected by rainfall than *A*. *albopictus* that have outdoor larval habitats [32]. In that regard, *A*. *aegypti* transmitted outbreaks might be less influenced by the rainfall than the *A*. *albopictus* transmitted outbreaks.

A significant positive auto-correlation was observed between dengue incidence and a rise in temperature prior to 9 months from the outbreak in the current study. However, generally in most parts of Sri Lanka only a little fluctuation in temperature has been observed throughout the year. Immediate temperature changes do not show any association with dengue outbreaks. This is further supported by a previous study done in three districts of Sri Lanka that led to the conclusion that weekly average temperatures do not significantly affect the dengue incidence. A study done in southern Taiwan showed that mean and maximum temperatures were negatively associated with dengue incidence in contrast to other regions of Taiwan where low temperature was the limiting factor for DENV transmission [33].

Although there are no significant temperature changes observed in Sri Lanka during the study period, districts like, Trincomalee and Hambantota with higher temperatures (29–35°C) and less rainfall had less dengue incidence. The harsh environmental conditions might be playing a role and the vectors might be less likely to establish in this environment. Yet outbreaks do occur with changing environmental conditions due to ongoing construction projects in the country including these districts. *A*. *aegypti* has been shown to transmit DENV when the temperature is above 20°C but not less than 16°C [34] and a positive correlation has been shown between temperature and the female vector abundance [4]. In addition, high temperatures may increase the frequency of blood feeding due to a rapid reduction in energy reserves in the vectors [34]. It is expected that global warming may facilitate the expanded distribution of DENV vectors in temperate regions like northern parts of North America and Europe [35]. This has become serious with the expanding distribution of *A*. *albopictus* to previously sterile areas [36].

The present study provides evidence for a mild positive auto-correlation between dengue incidence and high relative humidity prior to one-month although a significant negative auto-correlation was observed with high relative humidity prior to nine months from the outbreak. A power model between relative humidity and dengue incidence showed an inverse relationship based on a different study [27]. Conversely, relative humidity has been shown to negatively associate with dengue incidence as noted by Taiwanese study, which further explained that the mosquitoes generally survive longer in higher humid conditions and might bite more when they are water-stressed in low humid conditions and thus increasing the DENV transmission in the latter [36].

The prediction model in the current study (ARIMA) was used to predict data for a known data set which helps to assess the accuracy of the model. The data points aligned within the forecasted area suggests the usefulness of the model to predict future outbreaks. Prediction models can play a pivotal role for the effective use of resources for vector control in Sri Lanka. This study has covered 12 districts representing all 9 Provinces, hence, the model generated in this study is realistic to predict dengue outbreaks for the whole country.

### Conclusion

In conclusion, this study helps to map and evaluate the spatial and temporal distribution of dengue incidence using GIS in Sri Lanka. We have elucidated the association of geographical and climatic risk factors with dengue incidence. Thus GIS mapping using climatic risk factors with dengue incidence would aid health authorities and the government to take a look at high risk areas and make appropriate planning and implementation of control measures. Taking together the magnitude of the contribution of the environment, changing climatic conditions and the extreme manifestations of the climate change, the evolving DENV and the highly adapting vectors and their spread to the ever increasing dengue incidence (S2 Fig), health authorities must also pay attention to eliminate or minimize the contributing factors.

## Supporting Information

S1 Fig Distribution of DF/DHF cases in Sri Lanka from 2010–2015 (Curtsey: Sri Lankan Journal of Infectious Diseases. 2016;6(1):2–16).
(TIF)

S2 Fig Host, vector, virus and environmental factors for effective dengue control (Curtsey: Sri Lankan Journal of Infectious Diseases. 2016;6(1):2–16).
(TIF)
