# Supplementary figures and images for: Effect of Climatic Factors and Population Density on the Distribution of Dengue in Sri Lanka: A GIS Based Evaluation for Prediction of Outbreaks
Source: PLoS One. 2017 Jan 9;12(1):e0166806. doi: 10.1371/journal.pone.0166806 (PMC5222471; doi:10.1371/journal.pone.0166806)

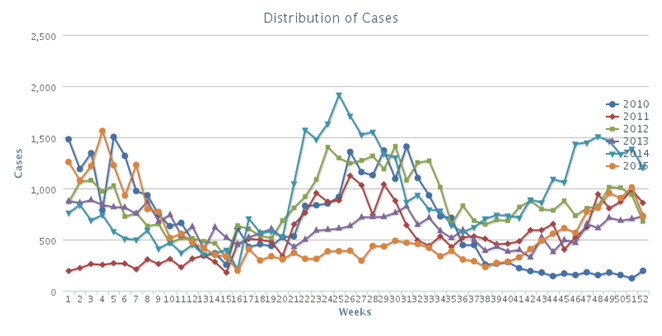

Supplement: S1 Fig — 2016;6(1):2–16). (TIF) [file pone.0166806.s001.tif]

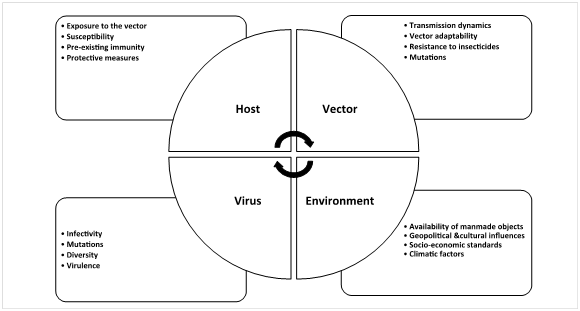

Supplement: S2 Fig — 2016;6(1):2–16). (TIF) [file pone.0166806.s002.tif]
